# Novel *Mucor* species (Mucoromycetes, Mucoraceae) from northern Thailand

**DOI:** 10.3897/mycokeys.84.71530

**Published:** 2021-11-01

**Authors:** Vedprakash G. Hurdeal, Eleni Gentekaki, Kevin D. Hyde, Thuong T.T. Nguyen, Hyang Burm Lee

**Affiliations:** 1 School of Science, Mae Fah Luang University, Chiang Rai, 57100, Thailand; 2 Center of Excellence in Fungal Research, Mae Fah Luang University, Chiang Rai, 57100, Thailand; 3 Environmental Microbiology Lab, Dept. of Agricultural Biological Chemistry, College of Agriculture and Life Sciences, Chonnam National University, Gwangju 61186, South Korea

**Keywords:** Molecular phylogeny, Mucorales, 1 geographical record, soil fungi, 2 new species

## Abstract

*Mucor* species are common fast-growing fungi found in soil. Two new species of *Mucor* and one new geographical record of *M.nederlandicus* were collected from northern Thailand and are described in this study. Evidence from morphophysiological data and phylogenetic analysis supports the introduction of the new taxa. Phylogenetic analysis based on the internal transcribed spacer (ITS) and large subunit of the nuclear ribosomal RNA (LSU) data showed that the new isolates cluster distinctly from other *Mucor* species with high or maximum bootstrap support. *Mucoraseptatophorus* is characterized by aseptate sporangiophores, globose columella, resistant and deliquescent sporangia, has sympodial, and monopodial branches and shows growth at 37 °C. It also differs from *M.irregularis* in having smaller sporangiospores, and larger sporangia. *Mucorchiangraiensis* has subglobose or slightly elongated globose columella, produces hyaline sporangiospores, and resistant and deliquescent sporangia. Furthermore, this species has wider sporangiophore, smaller sporangia and lower growth than *M.nederlandicus*. A detailed description of the species and illustrations are provided for the novel species.

## Introduction

Soil fungi play key roles in nutrient cycling and the functioning of terrestrial ecosystems (Torsvik and Øvreås 2002; Frąc et al. 2018; Peng et al. 2020; Zhou et al. 2020). To date, only a few fungal taxonomic studies are available from tropical forest soil (Sato et al. 2015; Amma et al. 2018; Cordeiro et al. 2020; Lima et al. 2020). Taxonomic studies on these groups are even scarcer in Southeast Asia despite the region harboring extraordinary genetic diversity (Amma et al. 2018). Up to 96% of fungi isolated from the northern part of Thailand are projected to be novel taxa and new species are regularly being discovered (Hyde et al. 2018; 2020). However, the diversity of non-Dikarya soil fungi in the country remains to be fully explored (Khuna et al. 2019; Hurdeal et al. 2021; Suwannarach et al. 2021).

Mucorales are cosmopolitan fungi commonly found in soil (Pawłowska et al. 2019; Muszewska et al. 2021). It is an early-diverging fungal group in a basal position with respect to Ascomycota and Basidiomycota in the fungal tree of life (Spatafora et al. 2016; Nicolás et al. 2020). The order comprises 55 genera with over 260 species (Walther et al. 2019; Cordeiro et al. 2020; Lima et al. 2020; Nicolás et al. 2020; Wijayawardene et al. 2020; Hurdeal et al. 2021). However, for the majority of these species, their ecological roles and geographical distributions are unknown. Mucoralean fungi are usually fast-growing and produce coenocytic or irregularly septate hyphae. Septae are usually formed to delimit reproductive structures but are not dispersed in regular intervals as in Dikarya fungi (Walther et al. 2019; Wagner et al. 2020). Characters including homothallism, formation of sporangiola, and the shape of the suspensors have been previously employed to describe mucoralean taxa (Walther et al. 2019). However, the application of molecular phylogenetics has transformed the taxonomy of Mucorales as it has revealed that some of the above-mentioned morphological characters are not taxonomically informative.

*Mucor* is the most species-rich genus within Mucorales commonly found in soil and dung. Its species comprise mainly saprobes, but also endophytes, parasites of plants and human pathogens causing mucormycosis (e.g. *M.irregularis*) (Mendoza et al. 2014; Lunge et al. 2015; Mishra et al. 2019; Wagner et al. 2020). *Mucor* was first described by Fresenius (1850) and has over 300 species cited in previous literature (Kirk 1986; Jacobs and Botha 2008; Nguyen et al. 2016; Lima et al. 2020). The suggested number of valid taxa varies from 50 to 91 species (Walther et al. 2019; Lima et al. 2020; Wijayawardene et al. 2020). *Mucor* species form fast-growing colonies characterized by simple or branched sporangiophores, non-apophysate and globose sporangia, deliquescent, and incrusted sporangial wall, and zygospores on opposed or tong-like suspensors. However, morphology-based classification of *Mucor* is highly debated. For example, previous literature used rhizoids to demarcate *Rhizomucor* from *Mucor* (Lima et al. 2020; Nguyen et al. 2020). Contrary to this, recent studies have revealed that some species of *Mucor* produce rhizoids (Lima et al. 2018; Nguyen and Lee 2020). *Mucor* comprises mesophilic species with some growing at high temperatures, but never at temperatures above 42 °C (Walther et al. 2019).

While studying the diversity of soil fungi in northern Thailand, two *Mucor* isolates differed morphologically and genetically from other known species. Using molecular phylogeny of ITS and LSU genetic markers along with morphological characterization, two new species of *Mucor* are proposed. Full description, taxonomic notes, photoplates, and phylogenetic trees are provided.

## Materials and methods

### Collections and field sites

Soil samples were collected from the provinces of Chiang Mai, and Chiang Rai, Thailand. Superficial organic matter (1–3 cm deep) was manually removed and a clean shovel was used to dig the soil. Samples were placed in a zip lock bag and stored at 4 °C until further use. The collecting site in Chiang Rai comprised a maple tree plantation and the sample was collected during the winter season in December 2019. The temperature in the province during this month is usually around 13.5 °C, while the annual rainfall is 2172 mm. The samples collected, consisted of peat and grainy soil. In Chiang Mai, moist peat soil was collected from a deciduous forest during the monsoon season in October 2019. During this season, the temperature is around 31 °C with an annual rainfall of 1108 mm.

### Isolation, culture and morphological studies

The dilution plating method was used for the isolation of fungal species (Senanayake et al. 2020). Soil samples were diluted to a ratio of 1:5 and 1:10 with sterilized distilled water. The mixture was shaken at 25 °C and 200 rpm for two hours using an incubator shaker. 100 μL of the suspension was then plated on agar plates. All Petri plates used herein were 90 mm. To maximize the number of fungi isolated, four different media were used: yeast malt extract (YMA) (yeast extract: 3g; malt extract: 3g; peptone: 5g; glucose: 10g; agar: 15g; distilled water: 1L), plate count agar (PCA) (enzymatic digest of casein: 5g; yeast extract: 2.5g; glucose: 1g; ; agar: 15g; distilled water: 1L), nutrient agar (NA) and modified choanephora agar (CH) (glucose: 3 g; pancreatic digest of casein: 2 g; KH_2_PO_4_: 1 g; MgSO_4_.7H_2_O: 0.5 g; thiamine HCl: 25 mg; agar: 20 g; distilled water: 1L) with or without benomyl. Benomyl (2 mg/L) was used to limit the growth of other fungi. Suspension of each soil sample was spread on four media using a flame sterilized rod spreader in triplicate. Plates were sealed and incubated at 20, 30, and 37 °C.

All plates were checked daily. Once fungal colonies were seen, the plates were screened using a microscope (400X) (Zeiss Primostar). Recovered strains were divided into morphotypes based on colony appearance and the presence of specific sporulation structures, when possible. A sterile straw was used to cut the fungal tips, which were transferred to a new agar plate. This process was done 2 or 3 days post-inoculation, before different fungal colonies overlapped on the plate. Growth experiments were performed using MEA media incubated at 8, 20, 25, 30, and 37 °C.

Once grown, the cultures were examined using a compound microscope (Nikon Eclipse Ni) and pictures were taken with a Nikon DS-RI2 digital camera. The cultures were maintained in 15% glycerol at 4 °C. Herbarium and type specimens were deposited in Mae Fah Luang University (MFLU) Herbarium, Chiang Rai, Thailand as inactive dried cultures. Ex-type living cultures were deposited in the Mae Fah Luang culture collection (MFLUCC), Chiang Rai, Thailand.

### DNA extraction and PCR amplification

Total genomic DNA was extracted from fungal mycelia using the Solg^TM^ Genomic DNA Prep Kit following the manufacturer’s instructions. Polymerase chain reaction (PCR) was used to amplify the partial fragments of internal transcribed spacer (ITS), and large subunit ribosomal RNA (LSU) using fungal-specific primers (Vilgalys and Hester 1990; White et al. 1990; De Hoog and Van Den Ende 1998). The PCR reaction mixture consisted of the fungal DNA, 14 μL dH_2_O, 1 μL AccuPower PCR PreMix (BioneerCorp., Daejeon, Korea) and 1.5 μL (5 pmol/μL) each of forward and reverse primers resulting in a final volume of 20 μL.

Amplification of the ITS and LSU fragments was performed using the following conditions: initial heat treatment of 5 min at 94 °C, 30 cycles with a denaturation step at 94 °C for 30 sec, annealing at 52 °C for 45 sec and an elongation step of 1 minute and 30 sec at 72 °C and a final elongation period of 7 minutes at 72 °C.

The PCR products were then purified using an Accuprep PCR Purification Kit (Bioneer). Sequencing was performed by Macrogen (South Korea) using an Applied Biosystems 3130XL DNA analyzer.

### Sequence and phylogenetic analysis

Raw reads were edited by removing ambiguous bases in the ends using BioEdit. The forward and reverse trimmed reads were assembled into contigs using SeqMan Version 7.1.0. Newly generated sequences were used as queries to perform blast searches against the nucleotide database in GenBank (Altschul et al. 1990). This was done to check for possible contamination and to find the closest taxa. The taxon sampling spans the diversity of the genus with the exception of some phylogenetically closely related species in the *M.circinelloides* complex. Information on each species was extracted from GenBank taxonomy and correlated with Species Fungorum (http://www.speciesfungorum.org/), Index Fungorum (http://www.indexfungorum.org/) and MycoBank (https://www.mycobank.org/) to determine the validity and current name of each taxon. Datasets of the ITS and LSU genetic markers were built. The software MAFFT Version 7 available on the online server https://mafft.cbrc.jp/alignment/server/ (Katoh and Toh 2008) was used to align the sequences and each matrix was trimmed using TrimAl (Capella-Gutiérrez et al. 2009). Genetic distances were calculated using the kimura2 parameter in MEGAX with the pairwise deletion gap option.

**Table 1.** Data used for phylogenetic analysis in this study and their corresponding GenBank accession numbers. Type, ex-neotype, ex-isotype, and ex-type strains are denoted by T, NT, IT, and ET, respectively. Sequences derived in this study are shown in bold letters.

Strain name	Voucher No.	ITS	LSU
*M.abundans* **^NT^**	CBS 388.35	JN206111	NG_063979
*M.abundans*	CBS 521.66	JN206110	JN206457
*M.aligarensis* **^T^**	CBS 993.70	NR_103634	NG_057920
*M.aligarensis*	NNIBRFG6255	MN267431	-
*M.amethystinus*	CBS 526.68	JN206015	JN206426.1
*M.amethystinus* **^T^**	CBS 846.73	JN206014	-
*M.amphibiorum* **^T^**	CBS 763.74	NR_103615	NG_057877
*M.ardhlaengiktus*	ATCC-MYA-4767	NR_111683	NG_042602
*M.ardhlaengiktus* **^ET^**	CBS 210.80	NR_152960	NG_069778
*M.atramentarius* **^T^**	CBS 202.28	MH854979.1	JN206418.1
*M.azygosporus* **^T^**	CBS 292.63	NR_103639	NG_057928
*M.bacilliformis* **^T^**	CBS 251.53	NR_145285	NG_057916
*M.bainieri* **^IT^**	CBS 293.63	NR_103628	JN206424
*M.caatinguensis* **^T^**	URM 7223	KT960377	KT960371
***M.chiangraiensis*^T^**	**MFLU 21–0079**	**MZ433253**	**MZ433250**
*M.chuxiongensis* **^T^**	CBS 14370	MG255732	MG255711
*M.circinatus*	URM 90063	KY008576	KY008571
*M.circinelloides*	B5–2	KT876701	-
*M.circinelloides*	CBS 108.16	JN205954	MH866163
*M.corticola*	CBS 362.68	JN206132	JN206449
*M.ctenidius* **^IT^**	CBS 293.66	MH858796	JN206417
*M.durus*	CBS 156.51	NR_145295	NG_057918
*M.endophyticus*	CBS 385.95	NR_111661	NG_057970
*M.exponens*	CBS 141.20	MH854686	JN206441
*M.falcatus*	CBS 251.35	NR_103647	NG_057931
*M.fluvii*	CNUFC-MSW21–1	MF667992	MF667995
*M.fluvii*	CNUFC-MSW21–2	MF667991	MF667996
*M.flavus* **^T^**	CBS 230.35	JN206061	JN206464
*M.fuscus*	CBS 132.22	JF723619	MH866227
*M.fuscus*	CBS 230.29	JN206204	FN650659
*M.fusiformis*	CBS 336.68	NR_111660	NG_057915
*M.genevensis* **^T^**	CBS 114.08	NR_103632	NG_057971
*M.genevensis*	CBS 535.78	-	-
*M.gigasporus*	CBS 566.91	NR_103646	NG_057926
*M.griseocyanus* **^T^**	CBS 116.08	NR_126136	NG_056283
*M.guiliermondii*	CBS 174.27	NR_103636	NG_057923
*M.guiliermondii*	ABTSJ72	KP790014	-
*M.heterogamus*	CBS 338.74	JN206169	JN206488
*M.hiemalis*	CBS 115.18	JN206127	-
*M.inaequisporus*	CBS 255.36	JN206177	NG_057929
*M.inaequisporus*	CBS 351.50	JN206178	MH868169
*M.indicus*	CBS 226.29	NR_077173	NG_057878
*M.irregularis* **^T^**	CBS 103.93	JN206150	NG_056285
*M.irregularis*	CBS 977.68	JX976259	JX976214
*M.irregularis*	CBS 700.71	JX976247	JN206450
*M.irregularis*	CBS 100164	JX976258	JX976213
*M.irregularis*	CBS 609.78	JX976260	JX976215
*M.irregularis*	TWS48Abf-e	MN629208	-
*M.japonicus* **^NT^**	CBS 154.69	JN206158	JN206446
*M.koreanus*	EML-QT1	KT936259	NG_068529
*M.koreanus*	EML-QT2	KT936260	KT936254
*M.laxorrhizus*	CBS 143.85	NR_103642	NG_057914
*M.lusitanicus* **^ET^**	CBS 108.17	JN205980	NG_056279
*M.luteus*	CBS 243.35	JX976254	NG_057969
*M.megalocarpus*	CBS 215.27	NR_145286	NG_057925
*M.merdicola* **^T^**	URM 7222	KT960374	KT960372
*M.merdophylus* **^T^**	URM 7908	MK775467	MK775466
*M.minutus* **^T^**	CBS 586.67	NR_152958	JN206463
*M.moelleri* **^T^**	CBS 406.58	NR_111659	MH869359
*M.mousanensis*	CBS 999.70	NR_103629	NG_057912
*M.mucedo*	CBS 542.66	JN206086	-
*M.mucedo*	CBS 987.68	JN206089	JN206480
*M.multiplex*	CBS 110662	NR_111662	NG_057924
*M.nederlandicus*	CBS 735.70	MH859923	MH871720
***M.nederlandicus***	**MFLU 21–0078**	**MZ433254**	**MZ433251**
*M.nidicola*	Isolate H13	KX375786	KX375769
*M.odoratus*	CBS 130.41	NR_145287	NG_057927
*M.orantomantidis* **^T^**	CNUFC-MID1–1	MH594737	MH591457
*M.parviseptatus*	CBS 417.77	JN206108	JN206453
*M.pernambucoensis* **^T^**	URM 7640	MH155323	MH155322
*M.piriformis*	CBS 169.25	NR_103630	NG_057874
*M.plasmaticus*	CBS 275.49	JN206078	JN206483
*M.plumbeus*	CBS 634.74	HM999955	HM849677
*M.prayagensis*	CBS 652.78	JN206189	JN206498
*M.pseudocircinelloides* **^T^**	CBS 541.78	JN206013.1	JN206431.1
*M.pseudolusitanicus* **^T^**	CBS 540.78	MF495059	NG_073591
*M.pseudolusitanicus*	CBS 543.80	MF495060.1	-
*M.racemosus*	CBS 115.08	JN206433	JN939201
*M.racemosus*	CBS 260.68	NR_126135	HM849676
*M.ramosissimus* **^NT^**	CBS 135.65	NR_103627	NG_056280
*M.rudolphii*	WU 35867	KT736104	-
*M.rudolphii*	WU 35869	NR_152977	-
*M.saturninus* **^T^**	CBS 974.68	NR_103635	JN206458
*M.septatum*	URM 7364	KY849814	KY849816
*M.silvaticus*	CBS 249.35	JN206122	JN206455
***M.aseptatophorus*** ^T^	**MFLU 21–0040**	**MZ433252**	**MZ433249**
*M.souzae*	URM 91186	KY992878	KY992879
*Mucor* sp.	MFLU 21–0082	MZ379497	MZ379500
*Mucor* sp.	P1	EU551186	
*Mucor* sp.	P2	FJ613116	FJ613117
*M.stercorarius*	CNUFC-UK2–1	KX839689	KX839685
*M.stercorarius*	CNUFC-UK2–2	KX839680	KX839682
*M.strictus*	CBS 100.66	JN206035	JN206477
*M.ucrainicus*	CBS 674.88	JN206192	JN206507
*M.ucrainicus*	CBS 221.71	MH860077	MT523853
*M.variicolumellatus* **^T^**	CBS 236.35	JN205979	JN206422.1
*M.variicolumellatus*	JMRC SF012536	MF495054.1	-
*M.variisporus*	CBS 837.70	NR_152951	NG_057972
*M.zonatus*	CBS 148.69	NR_103638	NG_057917
*M.zychae*	CBS 416.67	NR_103641	NG_057930
*B.dispersa*	CBS 195.28	JN206271	JN206530
*B.grandis* **^T^**	CBS 186.87	NR_103648	JN206527

Isolates and accession numbers determined in the current study are indicated in bold. ATCC: American Type Culture Collection, Virginia, United States; CBS: Centraalbureau voor Schimmelcultures, Utrecht, The Netherlands; CNUFC: Chonnam National University Fungal Collection, Gwangju, South Korea; JMRC: Jena Microbial Resource Collection, Jena Germany; MFLU: Mae Fah Luang University Herbarium, Chiang Rai, Thailand; URM: Departmento de Micologia of the Universidade Federal de Pernambuco, Recife, Brazil.

The nucleotide substitution models were evaluated for each genetic marker on the online CIPRES Portal (https://www.phylo.org/portal2) using the jModelTest2 on XSEDE and GTR + I + G was deemed as the best suited.

Maximum likelihood (ML) analysis was performed on the online CIPRES Portal using RAxML-HPC2 on XSEDE Version 8.2.12 with bootstrap support obtained from 1000 pseudoreplicates (Miller et al. 2010; Stamatakis 2014). The ML analysis was performed by partitioning the dataset according to the specific genetic marker. Bayesian inference (BI) analysis (Huelsenbeck and Ronquist 2001) was also performed on the online CIPRES Portal using MrBayes on XSEDE Version 3.2.7a. The analysis was conducted by running four simultaneous chains for 5,000,000 generations in two independent runs with a sampling frequency of 100 and temp parameter was adjusted to 0.05. At the end of the analysis the standard deviation of split frequency was less than 0.01 at which point convergence of the runs was declared.

## Results

### Phylogenetic analyses

The blast search against the NCBI database indicated that the strains belonged to *Mucor* as the majority of results were of the same genus with some being type species. The phylogenetic tree comprised 102 taxa including the strains isolated in this study. *Backuselladispersa* (CBS 195.28), and *B.grandis* (CBS 186.87) were used as outgroup taxa. After the removal of ambiguous positions, the ITS alignment contained 553 sites for ITS and that of LSU 1464 sites. The concatenated ITS-LSU alignment consisted of 2017 characters. The final concatenated matrix comprised 813 distinct alignment patterns and 39.49% of undermined characters or gaps. The tree topologies from ML and BI were mostly congruent. Phylogenetic analysis showed that the new strains formed distinct clades with maximum bootstrap support (BS). The isolate MFLU 21–0145 was sister to the clade formed by *Mucor* sp. MFLU 21–0082 and *Mucor* sp. TWS48Abf-e (BS:82/PP:0.97) and the three to *M.irregularis* (BS:100/PP:1.00). The MFLU 21–0079 strain was sister to the clade formed by *Mucor* sp. P1 and *Mucor* sp. P2 (BS:100/PP:1.00). The MFLU 21–0078 strain grouped with *M.nederlandicus* (BS:100/PP:1.00). The MFLU 21–0082 and MFLU 21–0078 clades grouped together (BS:100/PP:1.00) and as sister to *M.inaequisporus*, however this latter relationship is not strongly supported (CBS 255.36, CBS 351.50; PP: 0.95). The genetic distance between the novel taxa and their closely related taxa in the trimmed ITS gene region (578 bp for MFLU 21–0145 and *M.irregularis* group; 563 bp for MFLU 21–0079, MFLU 21–0078, and *M.inaequisporus* group) is shown in Table 2.

**Table 2.** Genetic distance (%) of the trimmed ITS region between the newly described *Mucor* species and their respective sister taxa. Distances were calculated using the Kimura2 parameter and gaps were considered as pairwise deletion.

Strains	% Genetic distance
	*M.aseptatophorus*MFLU 21–0145
*Mucor* sp. MFLU 21-0082	2.5
*Mucor* sp. TWS48Abf-e	1
*M.irregularis* CBS 977.68	3.5
*M.irregularis* CBS 103.93	4.5
*M.irregularis* CBS 609.78	4.5
*M.irregularis* CBS 100164	4
*M.irregularis* CBS 700.71	3
	*M.chiangraiensis*MFLU 21–0079
*Mucor* sp. P1	1.5
*Mucor* sp. P2	1
*M.nederlandicus*MFLU 21–0078	7.5
*M.nederlandicus* CBS 735.70	5.5
*M.nederlandicus* CBS 255.36	12.5
*M.inaequisporus* CBS 351.50	13.5

### Taxonomy

#### Mucor aseptatophorus

Taxon classificationFungiMucoralesMucoraceae

V.G. Hurdeal, E. Gentekaki, K.D. Hyde & H.B. Lee sp. nov.

72EB2AA7-9201-5907-BFCB-092026056741

 840562

Facesoffungi Number: FoF09923

Figs 1 , 2

##### Etymology.

Named after the aseptate sporangiophores produced by this species.

##### Holotype.

MFLU 21–0145

##### Gene sequences.

(ITS) MZ433252; (LSU) MZ433249

##### Diagnosis.

*Mucoraseptatophorus* is phylogenetically distinct from *M.irregularis*. In the phylogenetic analysis, *M.aseptatophorus* groups as sister to two *Mucor* sp. and all of them cluster as sister to the clade formed by *M.irregularis* strains with high bootstrap support. In contrast to *M.irregularis*, the ellipsoidal, cylindrical, or pyriform columella are not observed in *M.aseptatophorus*. Columella formed in the latter are globose. *Mucoraseptatophorus* has smaller sporangiospores (3.5–5 × 2–2.5 µm), slightly bigger sporangia, forms sympodial, and monopodial branching of sporangiospores and has a lower growth rate than *M.irregularis*. The species differs from *M.merdicola* and *M.nidicola*, by having smaller columella, sporangia and sporangiospore. Compared to *M.souzae*, sporangiophores in *M.aseptatophorus* are aseptate (below sporangia). Septation, when observed, is usually present at the branching point. Septae below the sporangia rarely observed.

**Figure 1.**
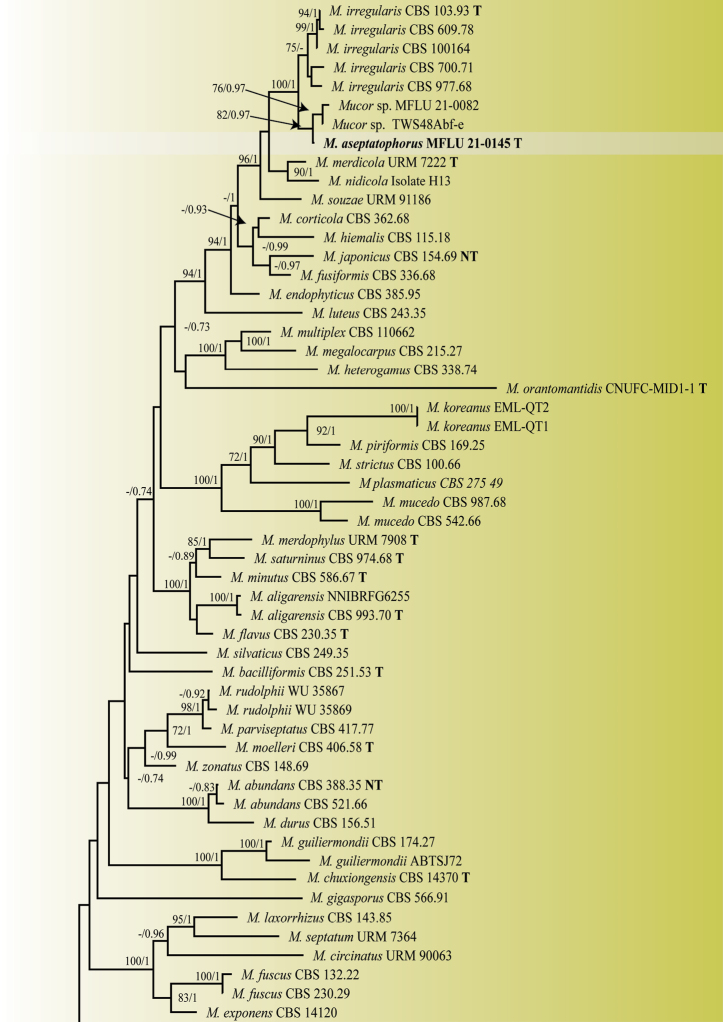
Maximum likelihood phylogram inferred from 102 taxa and 2017 characters based on ITS, and LSU matrix using GTR+G+I model and partition analysis. Maximum likelihood bootstrap support (≥ 70%) and Bayesian posterior probability (≥ 0.70) are indicated above the branches or near the nodes in this order. The tree is artificially rooted using *Backuselladispersa* (CBS 195.28), and *B.grandis* (CBS 186.87). The new species are in bold and the type species in the dataset are indicated using T. (-) represent bootstrap support lower than 70% or posterior probability lower than 0.70.

**Figure 1.**
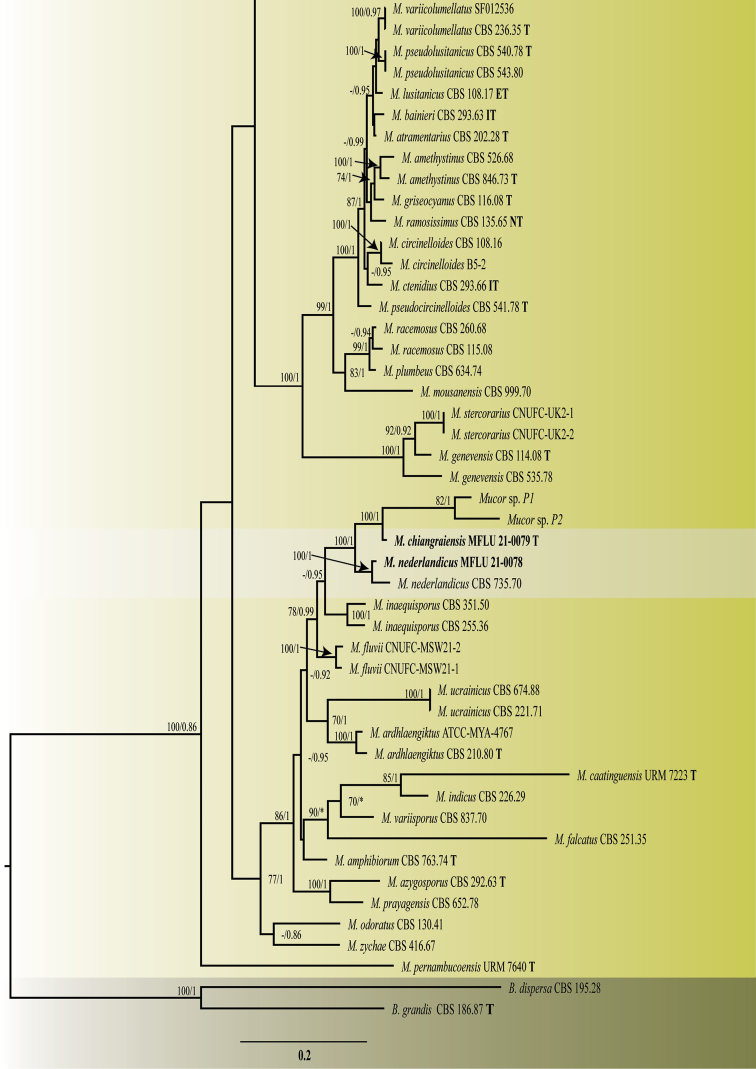
— continued.

##### Material examined.

Thailand. Chiang Mai Province, Omkoi District, Sop Khong, 17°45'25"N; 98°20'21"E, from soil, 24^th^ October 2019, collected by Oundhyalah Devi Padaruth, and isolated by Vedprakash Godadhar Hurdeal, ex-type living culture, MFLUCC 21–0040.

##### Description.

Asexual morph (based on cultures grown in MEA at 25 °C): Sporangiophores hyaline to pale brown, variable in length, erect, arising directly from the substrate, up to 17 µm in width (*x*– = 8.5 µm, *n* = 30), sympodial, and monopodial, with occasional circinate branches (mostly sympodial branches), and no septae observed. Sporangia 18–56.5 × 19–54 µm (*x*– = 41.5 × 41.5 µm, *n* = 40), globose to subglobose, smooth-walled, thick-walled and persistent, yellow to pale brown. Columellae 13–35 × 14–37.5 µm (*x*– = 19 × 20 µm, *n* = 40), globose, with very short collar, hyaline to pale brown, non-collapsing, smooth-walled. Sporangiospores 3.5–6 × 2–4 µm (*x*– = 4 × 3 µm, *n* = 70), mostly ellipsoidal, occasionally oval to globose, some irregular, hyaline. Chlamydospores and rhizoids present. Sexual morph not observed.

##### Culture characteristics.

Colonies on MEA reaching 62 mm diameter after 2 days of incubation at 25 °C. Colony white at first, becoming pale yellow with age; reverse pale yellow. Colony fully covers the Petri plate (90 mm) by the third day at 25 and 30 °C but does not reach the lid of the plate. At 20 °C, colony reaches a diameter of 70.5 mm after 3 days. Vertical growth is lower at 25 and 30 °C than at 20 °C. The colony does not reach the lid after 3 days. At 30 °C, sporulation is excellent, with branching of sporangiophore more frequent than in others. Monopodial branching more prominent but sympodial and dichotomous branches also observed. On PDA, cultures are white and pale brown in the middle with grey to pale brown sporangia. Colony reaching 64 mm diameter after 3 days of incubation at 25 °C. Optimal growth and excellent sporulation were observed on both MEA and PDA media at 30 °C. At 37 and 8 °C in MEA, growth is observed but with no sporulation. The colony reaches a diameter of 31 mm at 37 °C after 3 days. At 8 °C, the colony reaches a diameter of 14 mm after 9 days. Growth is observed at temperatures ranging from 8 to 37 °C.

##### Distribution.

Thailand.

#### Mucor chiangraiensis

Taxon classificationFungiMucoralesMucoraceae

V.G. Hurdeal, E. Gentekaki, K.D. Hyde & H.B. Lee sp. nov.

B4FF8CD2-577F-5D7C-ABD0-1AE1A5B9781D

 840564

Facesoffungi Number: FoF09924

Figs 1 , 3

##### Etymology.

The epithet refers to the province of Chiang Rai where the species was isolated.

##### Holotype.

MFLU 21–0079

##### Gene sequences.

(ITS) MZ433253; (LSU) MZ433250

##### Diagnosis.

In contrast to *M.nederlandicus*, this species produces smaller sporangia and has wider sporangiophores. The sporangia formed by *M.nederlandicus* are echinulate at maturity, while in *M.chiangraiensis* they are smooth-walled. This species mostly has subglobose or slightly elongated globose columellae while in *M.nederlandicus* they are mostly globose. Physiological data indicate that *M.nederlandicus* has a faster growth than *M.chiangraiensis* in MEA at 25 °C. Molecular phylogeny indicates that the newly isolated strain groups separately from *M.nederlandicus* and *M.inaequisporus*. Comparison with the protologue of *M.inaequisporus* indicates that *M.chiangraiensis* has smaller sporangia, columella and sporangiospores. The columellae in the latter are subglobose or slightly elongated globose columellae rather than commonly obovoid-pyriform or subspherical as in *M.inaequisporus*. Although the shape and size of sporangiospores are often variable in both species, the majority of spores of *M.chiangraiensis* are ellipsoidal. Sporangiospores are usually colorless and hyaline as compared to pale or greenish-yellow in *M.inaequisporus*.

**Figure 2.**
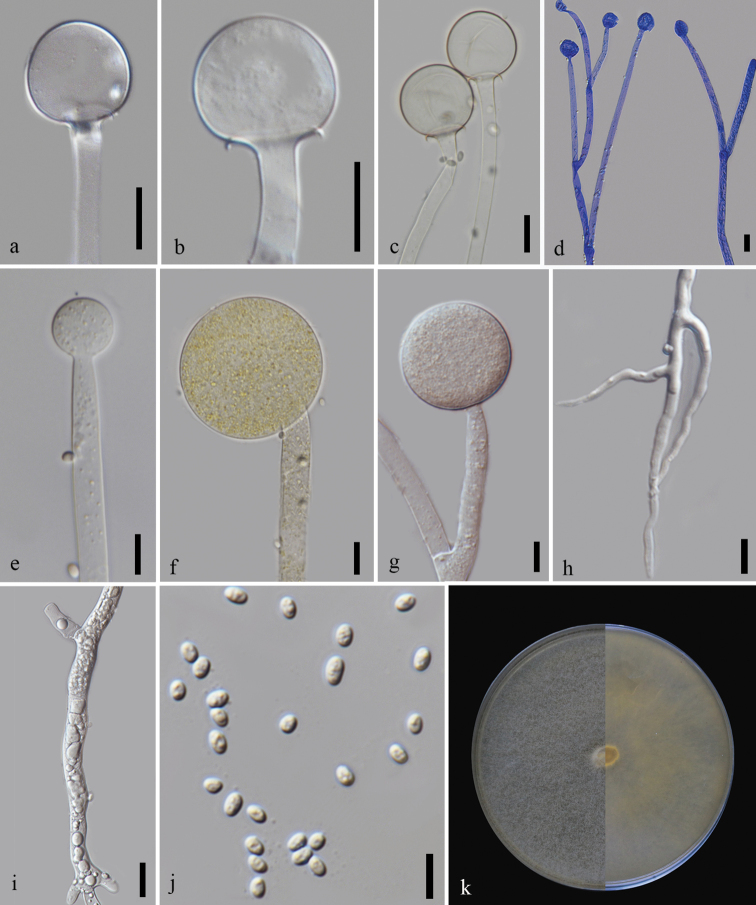
*Mucoraseptatophorus* (MFLU 21–0145) **a–c** columella with collar **d** branching of sporangiophores **e, f** developing sporangium **g** short sporangiophore with sporangium **h** rhizoids **i** granular content in mycelium **j** sporangiospores **k** front and reverse of the colony in MEA. Scale bars: 10 µm (**a–c, e–h, j**); 20 µm (**d, i**).

**Figure 3.**
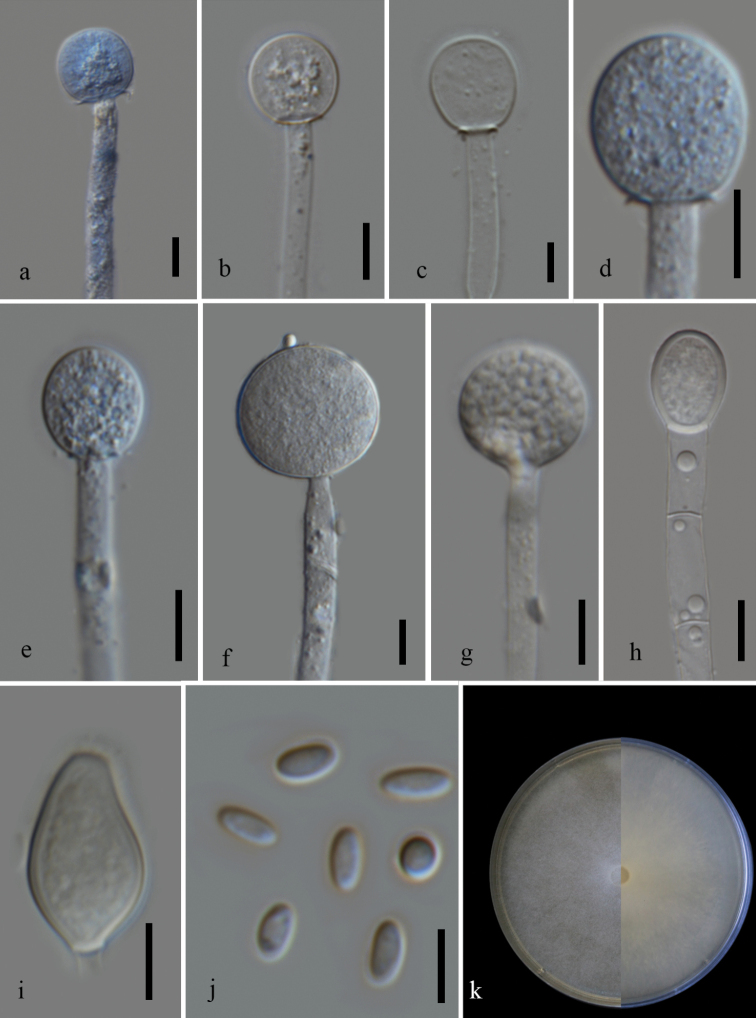
*Mucorchiangraiensis* (MFLU 21–0079) **a–e** columella with and without collars **f, g** sporangia **h–i** chlamydospores **j** sporangiospores **k** front and reverse of the colony grown in MEA. Scale bars: 10 µm (**a–j**).

##### Material examined.

Thailand. Chiang Rai Province, Doi Chang District, 19°49'9.984"N; 99°34'36.768"E, from soil, 7^th^ December 2019, collected and isolated by Vedprakash Godadhar Hurdeal, ex-type living culture, MFLUCC 21–0042.

**Description.** Asexual morph (based on cultures grown in MEA at 25 °C): Sporangiophores hyaline, up to 12 µm (*x*– = 6, *n* = 30) in diameter, erect, unbranched or sympodial branches. Hyphae irregular septate, with occasional hyphal bulges. Sporangia 18–40.5 × 18.5–40.5 µm (*x*– = 28.5 × 27.5 µm, *n* = 30), globose, wall resistant, deliquescent, hyaline to pale brown. Columellae 12.5–23.5 × 11.5–23 µm (*x*– = 19 × 17 µm, *n* = 30), subglobose or slightly elongated globose columellae, obovoid, ellipsoid and sometimes round to subglobose without or with short collar, hyaline to pale brown, smooth-walled. Sporangiospores 4–6.5 × 2–3.5 µm (*x*– = 5 × 2.5 µm, *n* = 50) µm, mostly ellipsoidal, oval, smooth-walled, hyaline. Chlamydospores abundant, intercalary, terminal, variable in shape and size. Rhizoids absent. Sexual morph not observed.

##### Culture characteristics.

Day-old cultures are white, cottony, floccose, have erect sporangiophores with hyphae reaching the lid of the Petri plate in MEA. The white color of the colony persists even after 6 days. At maturity, the colony reverse is white or pale yellow. On MEA at 25 °C, the colony reaches a diameter of 51 mm after 3 days of incubation. At 20 and 30 °C, the colony reaches a diameter of 47 mm and 44.5 mm respectively after 3 days. Sporulation is excellent at 20 to 30 °C. At 30 °C, mostly unbranched sporangiophores are observed with few sympodial branches. On PDA at 25 °C, colony reaches a diameter of 48 mm after 3 days of incubation. The front and reverse of the colony are white in both MEA and PDA. Growth is observed at temperatures ranging from 8 to 30 °C. Optimal growth and excellent sporulation were observed at 25 °C on both MEA and PDA media. No growth was observed at 37 °C. At 8 °C in MEA, the colony reached a diameter of 34 mm after 9 days but with no sporulation.

##### Distribution.

Thailand.

#### Mucor nederlandicus

Taxon classificationFungiMucoralesMucoraceae

Váňová, Česká Mykol. 45(3): 128 (1991)

ABE4E55F-58FA-5B9B-85D8-22A3D41AC21D

 354494

Figs 1 , 4

##### Gene sequences.

(ITS) MZ433254; (LSU) MZ433251

##### Material examined.

Thailand, Chiang Rai Province, Muang District, Mae Salong Nai, 20°12'38.0"N; 99°37'55.6"E, from soil, 10^th^ June 2020, collected by Bhavesh Raghoonundon, and isolated by Vedprakash Godadhar Hurdeal, living culture, MFLUCC 21–0045.

##### Description.

Asexual morph (based on cultures grown in MEA at 25 °C): Sporangiophores hyaline, up to 7.5 µm (*x*– = 5, *n* = 30) in diameter, undulate, occasionally curved, irregular septate near the base (1–2), unbranched, sympodial branches formed. Sporangia 20–47.5 × 20–45.5 µm (*x*– = 34 × 35.5 µm, *n* = 28), globose, smooth-walled, thick-walled, wall echinulate, deliquescent in mature sporangia, hyaline to pale brown. Columellae 12.5–23.5 × 12–22.5 µm ((*x*– = 16 × 15.5 µm, *n* = 30), mostly globose to subglobose, and sometimes oblong, obovoid, and rarely ellipsoid, without or with short collar, hyaline to pale brown, smooth-walled. Sporangiospores 3.5–6.5 × 2–4 µm (*x*– = 5 × 2.5 µm, *n* = 30) µm, mostly ellipsoidal, sometimes flattened on one side, oval or cylindrical, smooth-walled, hyaline with one or more granules. Chlamydospores abundant, intercalary, terminal, variable in shape and size. Rhizoids absent. Sexual morph not observed.

**Figure 4.**
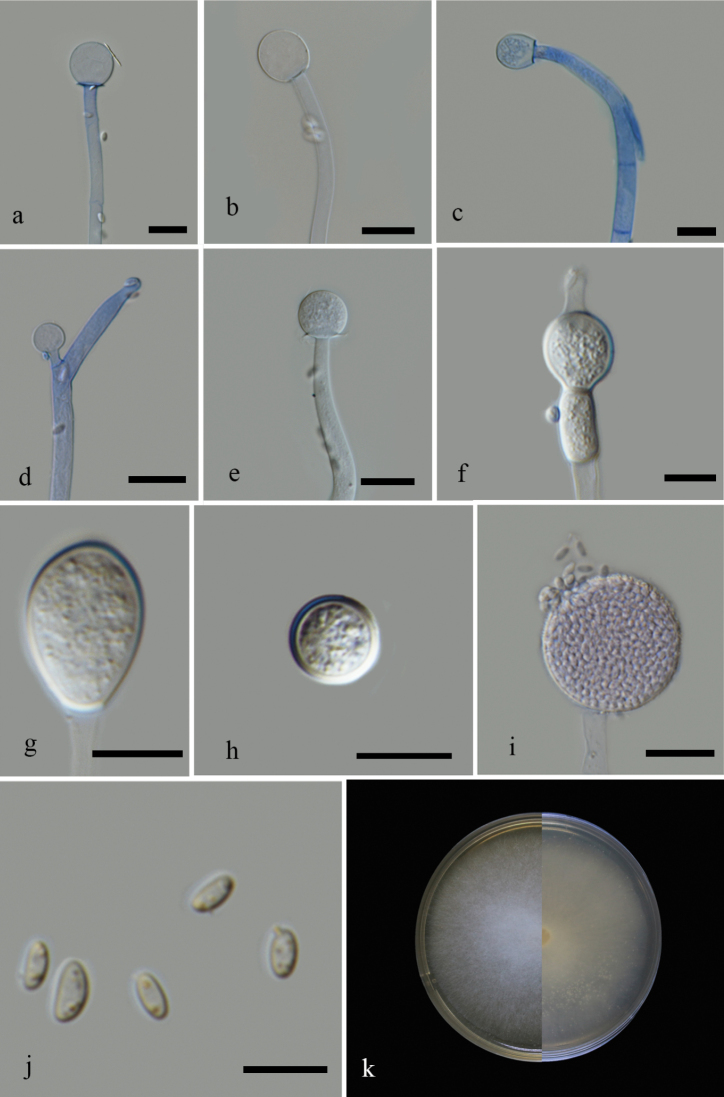
*Mucornederlandicus* (MFLU 21–007) **a-c** columella with highly reduced collar **d** sterile sporangium **e** columella with visible collar **f-h** chlamydospores **i** mature sporangia **j** sporangiospores **k** front and reverse of the colony in MEA. Scale bars: 20 µm (**a–e, i**); 10 µm (**f–h**).

##### Culture characteristics.

Colonies on MEA reaching a diameter of 60 mm after 3 days of incubation at 25 °C. At temperatures 20 and 30 °C, the colony diameter is 46 mm and 45.5 mm respectively after 3 days. Colony growth is observed at 8 to 30 °C with optimal growth at 25 °C. Unbranched or sympodial branching of sporangiophore are prominent at 30 °C. Colonies on PDA, reaching 51 mm after 3 days of incubation at 25 °C. In both culture media, colony from above and reverse: white or very pale yellow, and cottony. No growth was observed at 37 °C. At 8 °C, the colony reached a diameter of 21 mm after 9 days but with no sporulation in MEA.

##### Distribution.

Thailand, United Kingdom.

## Discussion

Thailand has extraordinary fungal diversity with many fungal species having been described from the northern provinces of the country (Hyde et al. 2018). The present study introduces two new species of *Mucor* and one new geographical record of *M.nederlandicus* from soil habitats in northern Thailand based on molecular and morphophysiological data. Many *Mucor* species have been isolated from soil and other substrates including postharvest crops, dung, and leaf litter (Lima et al. 2020; Nguyen et al. 2020). The newly-described *Mucor* species exhibit overall characteristic morphology observed in the genus, including branched sporangiophores and globose sporangia (Hoffmann et al. 2013; Walther et al. 2019). Commonly used morphological characters for species-level delimitation are the size and shape of structures, such as sporangiospores and columella (Chai et al. 2019; Lima et al. 2020). Morphological examination revealed that the newly isolated strains differ from their known sister taxa. This includes differences in the sporangia and columellae sizes. For example, *M.aseptatophorus* differs from its sister taxon *M.irregularis* in having smaller sporangiospores, but larger sporangia. Phenotypic characters comprise a pivotal aspect of modern fungal taxonomy. Nonetheless, some Mucorales groups display morphological plasticity, hence their taxonomy cannot be solely based on morphological features as these might not always be taxonomically informative.

In our phylogenetic analyses, placement of the new species was stable with high statistical support using both ML and BI methods of inference. Genetic distance analysis of the ITS region contributed further evidence to the introduction of the two novel *Mucor* species. The percentage nucleotide difference in the ITS genetic marker to the sister taxa is 2.5–4.5% for *M.aseptatophorus* and 1–13.3% for *M.chiangraiensis* (Table 2), exceeding the threshold for establishing new species (Walther et al. 2013; Jeewon and Hyde 2016).

Most studies focusing on *Mucor* species delimitation use ITS and LSU as these are the most widely available genetic markers (Madden et al. 2012; Lima et al. 2018; Chai et al. 2019; Lima et al. 2020). In the *Mucorcircinelloides* complex, protein-coding genes, such as the largest subunit of RNA polymerase II (*rpb1)* are also available for most species (Walther et al. 2019; Wagner et al. 2020). Several genetic markers, which have been traditionally used in the taxonomy of Dikarya fungi, cannot be applied in Mucorales due to the presence of paralogous and multiple-copy genes in the latter (Walther et al. 2019; Wagner et al. 2020). In general, the number of genetic markers necessary for species delimitation varies depending on the approach (Luo et al. 2018; Bhunjun et al. 2020). For instance, automatic barcode gap discovery can be used or best suited with single locus, while at least a couple of unlinked markers from independent loci are necessary for the genealogical concordance phylogenetic species recognition (GCPSR) concept (Laurence et al. 2014). Thus, only comparatively few studies use the latter (Taylor et al. 2000; Walther et al. 2019; Wagner et al. 2020). In the case of *Mucor*, the availability of only ITS and LSU for most taxa precludes the use of the GCPSR concept, as both markers belong to the same locus. Recently, phylogenomics approaches using hundreds of genes have started to emerge in an effort to delineate problematic fungal taxa (Vandepol et al. 2020). Nonetheless, in the case of *Mucor*, using ITS and LSU to delineate species has been adequate.

Herein, *M.aseptatophorus* showed growth at 37 °C. *Mucorirregularis*, which has close phylogenetic affinity to the new species also has the ability to grow at this temperature. *Mucorirregularis* is an opportunistic pathogen causing cutaneous mucoromycosis mostly in immunocompromised individuals (Lu et al. 2013; Xu et al. 2018; Soare et al. 2020). Thermotolerance in *Mucor* usually hints at pathogenic potential, however not all thermotolerant species are pathogens. Thus, the thermotolerant ability of *M.aseptatophorus* warrants further investigation in order to determine whether or not it is pathogenic.

In the last decade, at least 20 new *Mucor* species have been introduced and described from soil, freshwater, leaf litter, and dung habitats, indicating that we are nowhere near to discovering all taxa in the genus (Hyde et al. 2020). Information on the geographic distribution of *Mucor* has started to emerge. Its species have been isolated in Brazil, Great Britain, the USA, China, South Korea, Finland, Germany, and France, suggesting that *Mucor* has worldwide distribution occupying diverse habitats. Herewith, this study contributes to the study of *Mucor* in Thailand. As more species are being discovered, and the diversity of these organisms is being explored, the ecological roles of *Mucor* remain largely unknown and many habitats are yet to be explored. This highlights the need for more studies that explore the ecological importance, diversity, and accurate taxonomic classification of Mucorales.

## Supplementary Material

XML Treatment for Mucor aseptatophorus

XML Treatment for Mucor chiangraiensis

XML Treatment for Mucor nederlandicus
